# A mathematical model of circadian rhythms and dopamine

**DOI:** 10.1186/s12976-021-00139-w

**Published:** 2021-02-17

**Authors:** Ruby Kim, Michael C. Reed

**Affiliations:** Department of Mathematics, Duke University, 120 Science Drive, Box 90320, Durham, 27708 NC USA

**Keywords:** Circadian rhythms, Dopamine, Mathematical model

## Abstract

**Background:**

The superchiasmatic nucleus (SCN) serves as the primary circadian (24hr) clock in mammals and is known to control important physiological functions such as the sleep-wake cycle, hormonal rhythms, and neurotransmitter regulation. Experimental results suggest that some of these functions reciprocally influence circadian rhythms, creating a highly complex network. Among the clock’s downstream products, orphan nuclear receptors REV-ERB and ROR are particularly interesting because they coordinately modulate the core clock circuitry. Recent experimental evidence shows that REV-ERB and ROR are not only crucial for lipid metabolism but are also involved in dopamine (DA) synthesis and degradation, which could have meaningful clinical implications for conditions such as Parkinson’s disease and mood disorders.

**Methods:**

We create a mathematical model consisting of differential equations that express how the circadian variables are influenced by light, how REV-ERB and ROR feedback to the clock, and how REV-ERB, ROR, and BMAL1-CLOCK affect the dopaminergic system. The structure of the model is based on the findings of experimentalists.

**Results:**

We compare our model predictions to experimental data on clock components in different light-dark conditions and in the presence of genetic perturbations. Our model results are consistent with experimental results on REV-ERB and ROR and allow us to predict the circadian variations in tyrosine hydroxylase and monoamine oxidase seen in experiments. By connecting our model to an extant model of dopamine synthesis, release, and reuptake, we are able to predict circadian oscillations in extracellular DA and homovanillic acid that correspond well with experimental observations.

**Conclusions:**

The predictions of the mathematical model are consistent with a wide variety of experimental observations. Our calculations show that the mechanisms proposed by experimentalists by which REV-ERB, ROR, and BMAL1-CLOCK influence the DA system are sufficient to explain the circadian oscillations observed in dopaminergic variables. Our mathematical model can be used for further investigations of the effects of the mammalian circadian clock on the dopaminergic system. The model can also be used to predict how perturbations in the circadian clock disrupt the dopaminergic system and could potentially be used to find drug targets that ameliorate these disruptions.

## Background

The neurotransmitter dopamine (DA) is involved in learning, motivation, and the reward system [1, 2]. In addition, DA is associated with many psychiatric and neuropathological conditions. A primary symptom of Parkinson’s disease (PD) is loss of DA in the striatum caused by the death of cells in the substania nigra pars compacta (SNc) [3–5]. Disruption of DA dynamics is associated with schizophrenia, drug dependence and Tourette’s syndrome [6]. Furthermore, ongoing research links these physiological conditions and DA dynamics to circadian rhythms. Neuropsychiatric disorders such as schizophrenia are accompanied by disrupted circadian rhythms [7]. Mice with a particular circadian gene mutation display locomotor hyperactivity and memory impairment [8], and cocaine seeking behavior in mice is known to vary diurnally [9]. Our interest in this paper is to use mathematical modeling to understand the mechanisms by which the circadian clock affects the dopaminergic system.

Several recent experimental papers have investigated the connections between the DA system and the circadian clock. The core clock gene Bmal1 encodes the Brain and Muscle ARNT-Like 1 (BMAL1) protein, which is known to drive rhythmic clock gene expression. Preitner et al. [10] have demonstrated that Bmal1 is regulated by two downstream protein products, orphan nuclear receptors REV-ERB and retinoic acid-related orphan receptors (ROR). A REV-ERB agonist was used by Solt et al. [11] to shift the phase of all major circadian clock genes. Ikeda et al. [12] have shown that REV-ERB and ROR also modulate the expression levels of the dopamine D3 receptor (DRD3). Chung et al. [13] and Sleipness et al. [14] measured diurnal variations in the rate-limiting step in the synthesis of DA, the enzyme tyrosine hydroxylase (TH). Hampp et al. [15] showed that monoamine oxidase (MAO), which catabolizes DA, is influenced by the clock gene Bmal1, and Castañeda et al. [16] measured DA diurnal variation in different light and dark conditions. The purpose of this paper is to create a mathematical model in which all of these influences and regulations are made quantitative so that we can determine whether these influences are sufficient to explain the observed circadian behavior of the DA system.

**Fig. 1:**
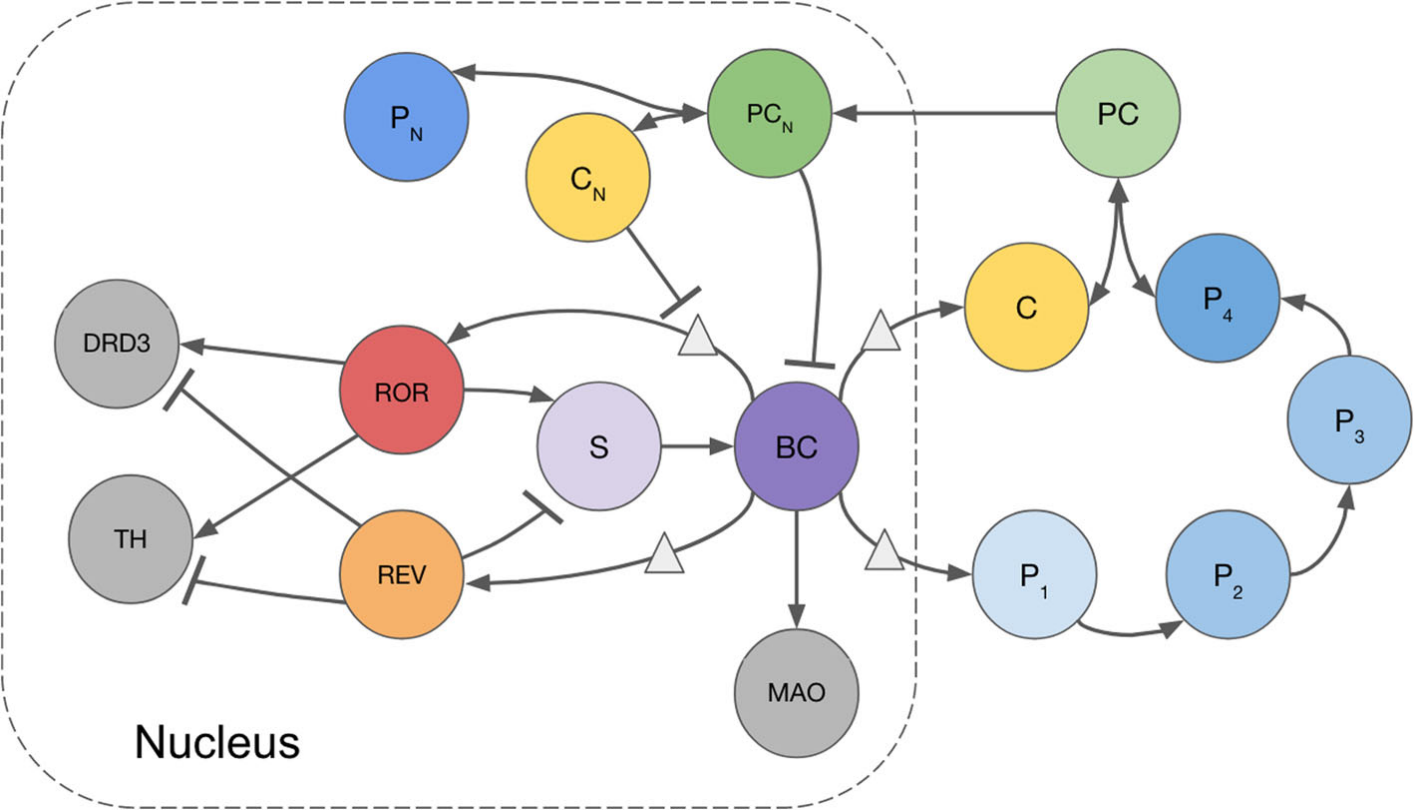
Schematic of Circadian-DA Model. Variables: BMAL1-CLOCK activator complex, *BC*; cytosolic Period (PER), *P*_*i*_ where *i* is the number of phosphorylations; cytosolic Cryptochrome (CRY), *C*; cytosolic PER-CRY complex, *PC*; nuclear PER-CRY complex, *PC*_*N*_; nuclear PER, *P*_*N*_; nuclear CRY, *C*_*N*_; retinoic acid receptor-related orphan receptor, *ROR*; orphan receptor REV-ERB, *REV*; Bmal1, *S*; Tyrosine hydroxylase, *TH*; Monoamine oxidase, *MAO*; Dopamine D3 receptor, *DRD*3

We develop a simple negative feedback loop model with three major interlocking systems: **(1)The core circadian clock** consists of BMAL1-CLOCK heterodimers that activate the transcription of Period (Per) and Cryptochrome (Cry) genes, which form heterodimer PER-CRY to inhibit their own transcription. Additional research on the detailed mechanisms of circadian rhythms reveals that the CRY protein independently acts as an even stronger transcriptional repressor [17, 18]. **(2)** In the **secondary feedback loop**, ROR activates the Bmal1 gene by binding to the ROR response element (RORE), a specific DNA sequence, in the Bmal1 promoter. REV-ERB represses Bmal1 after competing with ROR to bind to RORE in the Bmal1 promoter [10, 19]. **(3)** The **downstream influences** consist of the effects of the circadian clock on DA system components TH, MAO, and DRD3. These dopaminergic variables do have circadian rhythms [13–15], thought to be a result of the activation of MAO by BMAL1-CLOCK and the regulation of TH and DRD3 by REV-ERB and ROR [12, 20]. A schematic of the full model is provided in Fig. 1. Full details of the mathematical model, with references are given in the “Methods.”

There is a long history of contributions of modeling to the study of circadian rhythms: (1) Mathematical models at the single-cell level have been used to uncover new insights on circadian rhythms. Forger and Peskin [21] developed a highly detailed and robust model of the core mammalian clock circuitry. Leloup and Goldbeter [22] created a mammalian clock model which they used to simulate different light-dark conditions. Hong, Conrad, and Tyson [23] accounted for temperature compensation in a simple model of the core circadian negative feedback loop. (2) Another natural question is how diverse sets of cells (in, for example, the SCN) synchronize. This question connects directly to the large literature on coupled oscillators and systems with coupled limit cycles. Strogatz [24] reviewed 25 years of research on coupled limit cycles, particularly focusing on the Kuramoto model of coupled oscillators. Garcia-Ojalvo, Elowitz, and Strogatz [25] created a mathematical model of intercellular signaling and synchronization in biological oscillations. J. Kim and D. Forger have created several different mathematical models of the circadian clock and used the models to connect with and interpret experimental data [26–28].

In “The core circadian clock,” we demonstrate that our model of the core circadian clock agrees with major known properties of the clock including a 24 hour period length for clock components, the influence of light-dark, and responses to gene mutations. In “REV-ERB and ROR,” we use the model to investigate and interpret experimental results of Ikeda et al. [12] and Solt et al. [11] on the secondary feedback loop. In “DA system,” we study the influence of the core circadian clock and the secondary loop on the downstream DA variables *TH*, *MAO*, and *DRD*3, and compare our simulations to experimental data. Finally, in “Effect on extracellular dopamine and homovanillic acid,” we connect this mathematical model to the model of DA synthesis, release, and reuptake by Best et al. [29]. This enables us to predict the time course of DA in the extracellular space in the brain over a 24 hour period, and we compare our predictions to experimental data found by microdialysis [16].

## Methods

A schematic diagram of our model is given in Fig. 1 where each of the variables is described in the legend and full names are given in Table 1. The model consists of 16 differential equations and has three linked parts. The first is **the core circadian clock** that consists of a feedback loop containing *BC*, the successively phosphorylated Period proteins *P*_*i*_, *C*, the complex *PC* in the cytosol, and *PC*_*N*_,*C*_*N*_, and *P*_*N*_ in the nucleus. Light influences the clock by increasing the expression level of *P*_1_. This part of our model builds on the modeling in two papers by Kim and Forger [26, 27]. The second part is the **secondary feedback loop** containing *REV*, *ROR*, *S*, and *BC* that influences the core clock. And, the third part is the **downstream influences** that affect *TH*, *MAO*, and *DRD*3 in the dopaminergic system. All concentrations are in nanomolar (nM) and all rates are in nanomolar per hour (nM/hr). Model predictions were computed using a MATLAB differential equations solver, ode15s.

**Table 1:** Model variables

Variable	Description
*BC*	BMAL1-CLOCK; protein dimer that drives the transcription of
	core clock genes
*P*_*i*_	PERIOD protein (PER) with *i* phosphorylations in the cytosol
*C*	CRYPTOCHROME protein (CRY) in the cytosol
*PC*	PER-CRY protein dimer in the cytosol
*PC*_*N*_	nuclear PER-CRY protein dimer; inhibits BMAL1-CLOCK
*P*_*N*_	nuclear PER
*C*_*N*_	nuclear CRY; inhibits transcription driven by BMAL1-CLOCK
*ROR*	retinoic acid receptor-related orphan receptor (ROR)
*REV*	orphan nuclear receptor REV-ERB
*S*	Bmal1; circadian clock gene
*TH*	Tyrosine hydroxylase (TH); rate-limiting enzyme in dopamine
	synthesis
*MAO*	Monoamine oxidase (MAO); catalyzes dopamine degradation
*DRD*3	Dopamine receptor D3 (DRD3)

### The core circadian clock

*P*_1_ through *P*_4_ in the model represent the phosphorylation steps of PER, beginning with *P*_1_ being activated by free BMAL1-CLOCK (*BC*), which can bind to repressor PER-CRY (*PC*_*N*_). We write free *BC* as

1 $$ BC_{fr} = f(BC,PC_{N},K_{d})  $$

where the function *f* is defined by

2 $$ f(P,I,D) = \frac{P-I-D+\sqrt{(P-I-D)^{2}+4DP}}{2P}.   $$

The function *f* is used to describe protein sequestration [30]. *f*(*P*,*I*,*D*) is the proportion of free protein *P* (out of total *P*), which can bind to an inhibitor *I* to become inactive. This protein sequestration model was used successfully in [26] to model the inhibition of *BC* by *PC*_*N*_. The production rates of *P*_1_ and CRY (*C*) in the cytoplasm increase with *BC*_*fr*_. After PER goes through multiple phosphorylation steps, *P*_4_ binds to *C*, re-enters the nucleus as *PC*_*N*_, and represses the transcription of the Per and Cry genes.

To model the repression by nuclear CRY (*C*_*N*_) of clock components activated by *BC*, we use a function $\mathcal {F}(C_{N})$ that decreases with *C*_*N*_.

3 $$ \mathcal{F}(C_{N}) = \frac{\rho_{c}}{(1+k_{c} C_{N})^{n_{c}}}  $$

*ρ*_*c*_,*k*_*c*_, and *n*_*c*_ are parameters. *ρ*_*c*_ increases the activation of *P*_1_ as it gets larger, and *k*_*c*_ and *n*_*c*_ decrease the activation as they get larger; the values for these parameters are in Table 2. As detailed kinetics about the repression by CRY are not yet known, we had to choose reasonable values for the parameters, based on data in [17, 18]. A detailed discussion about parameter selection is provided in “Validating the model.” The equations for our core oscillator are given below, and parameter values provided in Table 2. The parameters for this part of our model were chosen so that the behavior of the core circadian clock would be consistent with [26, 27].

4 $$\begin{array}{*{20}l} \frac{dP_{1}}{dt} &=& r_{1} \mathcal{F}(C_{N}) BC_{fr} - r_{2} P_{1}  \end{array} $$

**Table 2:** Model parameter values

Parameter	Value	Reference
Core circadian clock		
*K*_*d*_	0.02	[26, 27]
*ρ*_*c*_	3	[17, 18]
*k*_*c*_	0.5	[17, 18]
*n*_*c*_	3	[17, 18]
*r*_1_	5	[26, 27]
*r*_2_	0.45	[26, 27]
*r*_3_	0.45	[26, 27]
*r*_4_	0.45	[26, 27]
*m*_*c*_	0.5	[26, 27]
*d*_4_	0.6	[26, 27]
*τ*_*c*_	0.5	[26, 27]
*r*_5_	5	[26, 27]
*d*_5_	0.1	[26, 27]
*d*_6_	0.12	[26, 27]
*r*_6_	0.75	[26, 27]
*d*_7_	0.2	[26, 27]
*τ*_*n*_	0.1	[26, 27]
*m*	0.5	[26, 27]
*d*_*p*_	0.25	[26, 27]
*d*_*c*_	0.2	[26, 27]
*β*_*bc*_	0.1	[26, 27]
*d*_*bc*_	0.1	[26, 27]
*ξ*	$\frac 23$	[26, 27]
Secondary loop		
*n*	2	[12, 31]
*κ*_*rev*_	0.2	[12, 31]
*κ*	1.5	[12, 31]
*ρ*_*s*_	1	[12]
*k*_*s*_	0.5	[12]
*ε*_*s*_	0	[12]
*n*_*s*_	5.3	[12]
*α*_*s*_	3.7	[12]
*κ*_*s*_	1	[12]
*r*_*rev*_	1.5	[12]
*d*_*rev*_	0.5	[12]
*b*_*ror*_	0.1	[12]
*r*_*ror*_	1.8	[12]
*d*_*ror*_	0.25	[12]
*β*	0.9	[12, 26]
*d*_*s*_	3	[12, 26]
DA elements		
*ρ*_*th*_	1	[13]
*k*_*th*_	10	[13]
*ε*_*th*_	0.4	[13]
*n*_*th*_	1	[13]
*α*_*th*_	1.23	[13]
*κ*_*th*_	1	[13]
*b*_*th*_	0.85	[13]
*d*_*th*_	5.6	[13]
*r*_*m*_	3	[15]
*d*_*m*_	0.016	[15]
*ρ*_*dr*_	1	[12]
*k*_*dr*_	10	[12]
*ε*_*dr*_	0.4	[12]
*n*_*dr*_	1	[12]
*α*_*dr*_	0.53	[12]
*κ*_*dr*_	10	[12]
*b*_*dr*_	0.3	[12]
*d*_*dr*_	3	[12]

5 $$\begin{array}{*{20}l} \frac{dP_{2}}{dt} &=& r_{2} P_{1} - r_{3} P_{2} \end{array} $$

6 $$\begin{array}{*{20}l} \frac{dP_{3}}{dt} &=& r_{3} P_{2} - r_{4} P_{3} \end{array} $$

7 $$\begin{array}{*{20}l} \frac{dP_{4}}{dt} &=& r_{4} P_{3} + m_{c} PC - d_{4} P_{4} - \tau_{c} P_{4} C \end{array} $$

8 $$\begin{array}{*{20}l} \frac{dC}{dt} &=& r_{5} \mathcal{F}(C_{N}) BC_{fr} + m_{c} PC - d_{5} C - \tau_{c} P_{4} C \end{array} $$

9 $$\begin{array}{*{20}l} \frac{dPC}{dt} &=& \tau_{c} P_{4} C - d_{6} PC - m_{c} PC \end{array} $$

10 $$\begin{array}{*{20}l} \frac{dPC_{N}}{dt} &=& r_{6} PC - d_{7} PC_{N} + \tau_{n} P_{N} C_{N} - m PC_{N} \end{array} $$

11 $$\begin{array}{*{20}l} \frac{dP_{N}}{dt} &=& m PC_{N} - \tau_{n} P_{N} C_{N} - d_{p} P_{N} \end{array} $$

12 $$\begin{array}{*{20}l} \frac{dC_{N}}{dt} &=& m PC_{N} - \tau_{n} P_{N} C_{N} - d_{c} C_{N} \end{array} $$

13 $$\begin{array}{*{20}l} \frac{dBC}{dt} &=& \beta_{bc} S - d_{bc} BC \end{array} $$

### REV-ERB and ROR

To model the production of *REV* and *ROR* as a function of *BC*_*fr*_, we rely on data in [12] and [31]. Experimental data [12] suggests that *REV* and *ROR* peak at the same time, with *REV* displaying larger fold changes. We create terms $\mathcal {G}_{1}$ and $\mathcal {G}_{2}$ for the production rates of *REV* and *ROR*.

14 $$\begin{array}{*{20}l} \mathcal{G}_{1}(BC_{fr}) &=& \frac{(BC_{fr})^{n}}{(\kappa_{rev})^{n}+(BC_{fr})^{n}} \end{array} $$

15 $$\begin{array}{*{20}l} \mathcal{G}_{2}(BC_{fr}) &=& \frac{BC_{fr}}{\kappa+BC_{fr}} \end{array} $$

To model the impact of *REV* and *ROR* on the clock, we create an intermediate step *S*, which can be thought of as Bmal1. REV-ERB and ROR compete to bind to the RORE sequence of the Bmal1 promoter, thus we have *REV* inhibit *S* and *ROR* activate *S* with competition for binding. Ikeda et al. [12] suggest that *REV* and *ROR* levels peak at the same time, with *REV* having a larger impact than *ROR* during peak levels. When they are not at their peak levels, *ROR* activates *S*. We use Eq. (2) to write *f*(*S*,*REV*,*ε*_*s*_) as the percentage of free *S* after *REV* binding, with dissociation constant *ε*_*s*_. Since 1−*f*(*S*,*REV*,*ε*_*s*_) is the percentage of *S* bound to *REV*, we write the repression term $\mathcal {R}_{s}$ as

16 $$ \mathcal{R}_{s}(S,REV) = \frac{\rho_{s}}{\left(1+k_{s}\left(1-f\left(S,REV,\epsilon_{s}\right)\right)\right)^{n_{s}}}  $$

so if *REV* binds more to *S*, then $\mathcal {R}_{s}$ decreases. Following this idea, we choose to have the activation term $\mathcal {A}_{s}$ be dependent on the percentage of free *S* and amount of *ROR*.

17 $$ \mathcal{A}_{s}(S,REV,ROR) = \alpha_{s} f(S,REV,\epsilon_{s}) \frac{ROR}{ROR+\kappa_{s}}  $$

We choose a simple equation for the change in *S*, by adding a basal production rate *β*, repression and activation terms $\mathcal {R}_{s}$ and $\mathcal {A}_{s}$, and a degradation term proportional to the amount of *S*. We chose parameter values such that the behavior of *S* would agree with the findings in [12, 26]. The equations for *REV*, *ROR*, and *S* are given below.

18 $$\begin{array}{*{20}l} {}\frac{dREV}{dt} &=& r_{rev} \mathcal{G}_{1}(BC_{fr}) \mathcal{F}(C_{N}) - d_{rev} REV \end{array} $$

19 $$\begin{array}{*{20}l} {}\frac{dROR}{dt} &=& b_{ror} + r_{ror} \mathcal{G}_{2}(BC_{fr})\mathcal{F}(C_{N}) - d_{ror} ROR \end{array} $$

20 $$\begin{array}{*{20}l} {}\frac{dS}{dt} &=& \beta + \mathcal{R}_{s}(S,REV) + \mathcal{A}_{s}(S,REV,ROR) - d_{s} S \end{array} $$

### Effect on the DA system

Tyrosine hydroxylase (TH) is the rate-limiting enzyme for the synthesis of DA. Evidence shows that TH expression is modulated by REV-ERB and ROR by competitive binding to the RORE element in the TH promoter [20]. We rely on data in [13] to model the repression and activation of *TH* by *REV* and *ROR* with $\mathcal {R}_{th}$ and $\mathcal {A}_{th}$, respectively.

21 $$\begin{array}{*{20}l} &&\mathcal{R}_{th}(TH,REV) \\ &&\qquad= \frac{\rho_{th}}{\left(1+k_{th}\left(1-f\left(TH,REV,\epsilon_{th}\right)\right)\right)^{n_{th}}} \end{array} $$

22 $$\begin{array}{*{20}l} &&\mathcal{A}_{th}(TH,REV,ROR)\\ &&\qquad= \alpha_{th} f(TH,REV,\epsilon_{th}) \frac{ROR}{ROR+\kappa_{th}} \end{array} $$

Monoamine oxidase (MAO) is involved in the degradation of DA, and is known to be activated by BMAL1-CLOCK [20]. We choose the production term of *MAO* to be directly proportional to $\mathcal {F}(C_{N})BC_{fr}$, as in the production terms of $\frac {dP_{1}}{dt}$ and $\frac {dC}{dt}$. The parameters *r*_*m*_ and *d*_*m*_ were chosen so that *MAO* behaves similarly to the data in [15].

23 $$\begin{array}{*{20}l} \frac{dTH}{dt} &=& b_{th} + \mathcal{R}_{th}(TH,REV) + \mathcal{A}_{th}(TH,REV,ROR)\\&& - d_{th} TH \end{array} $$

24 $$\begin{array}{*{20}l} \frac{dMAO}{dt} &=& r_{m} \mathcal{F}(C_{N}) BC_{fr} - d_{m} MAO \end{array} $$

The dopamine receptor D3 (DRD3), which plays an important role in cognition [32], is thought to be influenced by the core circadian clock, like TH, through REV-ERB dependent inhibition and ROR dependent activation [20]. We rely on data in [12] to model the repression and activation of *DRD*3 by *REV* and *ROR* with $\mathcal {R}_{dr}$ and $\mathcal {A}_{dr}$, respectively.

25 $$ \begin{aligned} \mathcal{R}_{dr}(DRD3,REV) &= \frac{\rho_{dr}}{\left(1+k_{dr}\left(1-f\left(DRD3,REV,\epsilon_{dr}\right)\right)\right)^{n_{dr}}}\\ \mathcal{A}_{dr}(DRD3,REV,ROR) &= \alpha_{dr} f(DRD3,REV,\epsilon_{dr}) \frac{ROR}{ROR+\kappa_{dr}} \end{aligned}  $$

In our model, the differential equation for *DRD*3 resembles that of *TH*.

26 $$ \begin{aligned} \frac{dDRD3}{dt} \!&=\! b_{dr} + \mathcal{R}_{dr}(DRD3,REV) + \mathcal{A}_{dr}(DRD3,REV,ROR)\\& - d_{dr} DRD3 \end{aligned}  $$

We connect our mathematical model to the extant mathematical model of DA synthesis, release, reuptake and control by autoreceptors created by Best et al. [29]. A schematic diagram of the model of Best et al. [29] is shown in Fig. 6a. The variables are in the pink boxes and the biochemical reactions and transport velocities are indicated by arrows. Full names are given in the figure legend. The corresponding mathematical model, including the differential equations, rate constants and Michaelis constants, is described completely in the Methods section of [29].

The model in this paper has as output the expression levels of TH and MAO as functions of time over a 24 hour period with light during the first 12 hours and dark during the second 12 hours. The functions *ETH*(*t*) and *EMAO*(*t*) are these expression levels scaled so that the maximum expression level is 1. In the model by Best et al. [29], we multiply the *V*_*max*_ of TH by *ETH*(*t*) and the *V*_*max*_ of MAO by *EMAO*(*t*) to compute the time courses of all the variables.

### REV-ERB agonist

Experiments using a synthetic REV-ERB agonist suggest that the agonist may induce phase shifts in major clock protein rhythms under 12hr light 12hr dark conditions [11]. We introduce another variable *Ag* for the amount of REV-ERB agonist over time. In [11], experimentalists reported that the agonist *Ag* decays exponentially to a negligible amount in 24 hours. Generally, a drug is considered to be negligible after 5 half-lives [33]. Therefore, we take the half-life of *Ag* to be 4.8 hours, so that *Ag* undergoes 5 half-lives in 24 hours. Then

27 $$ \frac{dAg}{dt} = -\frac{\ln(2)}{4.8}Ag  $$

In the REV-ERB agonist simulations, the model equations for the repression term $\mathcal {R}_{s}$ and activation term $\mathcal {A}_{s}$ in $\frac {dS}{dt}$ are adjusted so that *Ag* acts like *REV*.

28 $$ \frac{dS}{dt} = \beta + \mathcal{R}_{s}(S,REV+Ag) + \mathcal{A}_{s}(S,REV+Ag,ROR) - d_{s} S  $$

We chose a single injection of *Ag* to be double the *REV* peak value to exaggerate the effects of the agonist. This value could be adjusted as appropriate.

### Light input

The mechanisms of circadian entrainment to light are still not fully understood. Multiple studies have shown that PER expression is induced by light, likely as a result of increased neural activity [34–36]. This idea was used in [21] to incorporate light into their detailed model of the main mammalian circadian clock components. We incorporate light input by using a sinusoidal function *ℓ*(*t*) where $\xi =\frac 23$ with period 24hr to adjust the transcription rate of *P*_1_ so that it varies diurnally.

29 $$ \ell(t) = \xi \sin\left(\frac{\pi}{12}t\right)+1  $$

so Eq. 4 is replaced by

30 $$ \frac{dP_{1}}{dt} = r_{1}\ell(t) BC_{fr} \mathcal{F}(C_{N}) - r_{2} P_{1}  $$

### Validating the model

The purpose of the model we have constructed is to provide a platform for computational experiments to test hypotheses about how the mammalian circadian clock affects the DA system. We explain in detail in the Discussion why the sparseness and variability of the data precludes finding a “correct” set of parameters. Indeed, we expect that parameters will vary between species, between animals of the same species, and between different brain regions. There is almost no information on the parameters in the experimental literature. To choose the parameters in Table 2, we experimented with the parameters as follows. We first constructed the **core circadian clock** part of the model and experimented with different parameter choices and compared to data in the literature until we were satisfied that the core clock model has the key features of the circadian clock that have been established by the research community. We then added **the secondary feedback loop** and again experimented by trial and error with the parameters in that part until we were satisfied that the model has the qualitative behavior of REV and ROR seen in experiments. Then we added the **downstream influences** of REV and ROR and again experimented with the parameters until we had good qualitative fits of the experimental data on TH, MAO, and DRD3 expression. These parameters remain the same for all model curves in the paper except as indicated in the text. We did not use any curve fitting programs. For the experiments in “Effect on extracellular dopamine and homovanillic acid,” we put the variable expression levels that we compute for TH and MAO into the model of dopamine synthesis and release [29] and allowed that model, with no changes in its parameters, to compute the time course of extracellular DA and HVA.

**Fig. 2:**
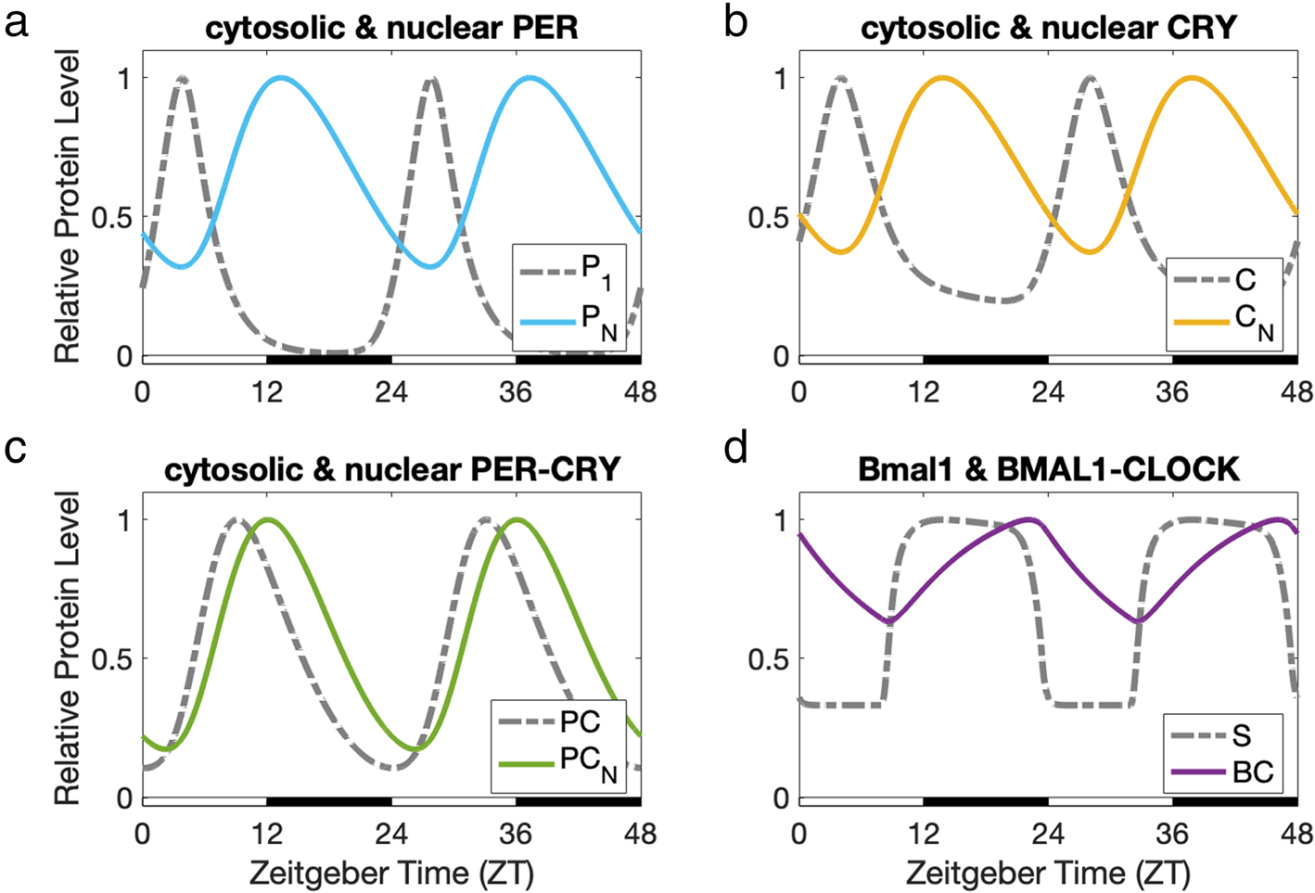
Core clock protein levels, relative to their peak values. The peaks of cytosolic PER (*P*_1_) and cytosolic CRY (*C*) (dashed gray curves in panels **a** and **b**) follow shortly after the peaks of BMAL1-CLOCK (*BC*) (solid purple curve in panel **d**). The delays between *P*_1_ and nuclear PER (*P*_*N*_), and between *C* and nuclear CRY (*C*_*N*_), are around 9 hours (Panels **a** and **b**). *P*_*N*_ and *C*_*N*_ reach maximum expression at the beginning of dark, consistent with experimental data [38]. *P*_*N*_ and *C*_*N*_ bind and form nuclear PER-CRY (*PC*_*N*_), which inhibits *BC* through sequestration. Thus, when *PC*_*N*_ peaks at 12 hours Zeitgeber time (ZT12), the expression levels of *P*_1_ and *C* are significantly reduced. Additionally, *C*_*N*_ directly inhibits the production of *P*_1_ and *C* during night, as proposed in [17]

**Fig. 3:**
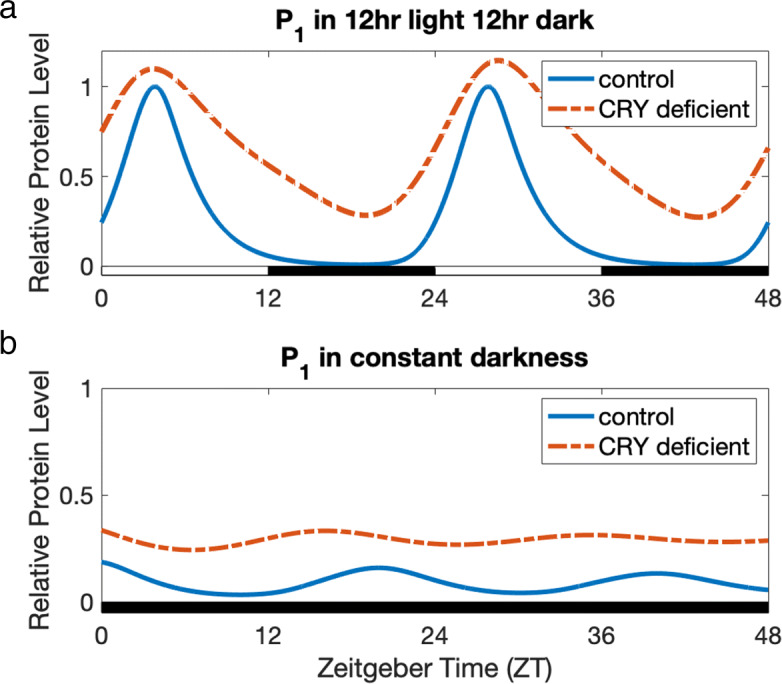
Impact of CRY deficiency on *P*_1_ in 12hr light 12hr dark (Panel **a**) and in constant darkness (Panel **b**). **a** *P*_1_ in 12hr light 12hr dark. The expression level of cytosolic PER (*P*_1_) in the model under 12hr light 12hr dark (blue curve) and the expression of *P*_1_ in the CRY deficient model under 12hr light 12hr dark (dashed orange curve) are graphed relative to the peak value of *P*_1_ under 12hr light 12hr dark (blue curve). We model CRY deficiency by reducing the production rate of CRY by 95%. In 12hr light 12hr dark conditions, CRY deficiency results in nearly diurnal oscillations and elevation in the expression level of *P*_1_, as found in mouse SCN experiments [40, 41]. **b** *P*_1_ in constant darkness. The expression level of *P*_1_ in the model under constant darkness (blue curve) and the expression of *P*_1_ in the CRY deficient model under constant darkness (dashed orange curve) are graphed relative to the peak value of *P*_1_ under 12hr light 12hr dark (Panel **a**, blue curve). It is known that Per gene expression is light-induced [34–36]. In constant darkness, *P*_1_ expression is still rhythmic, but greatly reduced in comparison to expression levels under 12hr light 12hr dark conditions. This is consistent with data for mice previously entrained to a 12hr light 12hr dark cycle, and then placed under constant darkness [11, Figure 2c]. Our model also displays a shorter period length in constant darkness, as observed in the mouse circadian oscillator [39]. Our model predicts that under constant darkness and CRY deficiency, the circadian oscillations of *P*_1_ (dashed orange curve) will be disrupted and nearly constant. This is consistent with data that have shown immediate arrythmicity and near constant PER expression levels in CRY null mutant mice under constant darkness [40, 41]

**Fig. 4:**
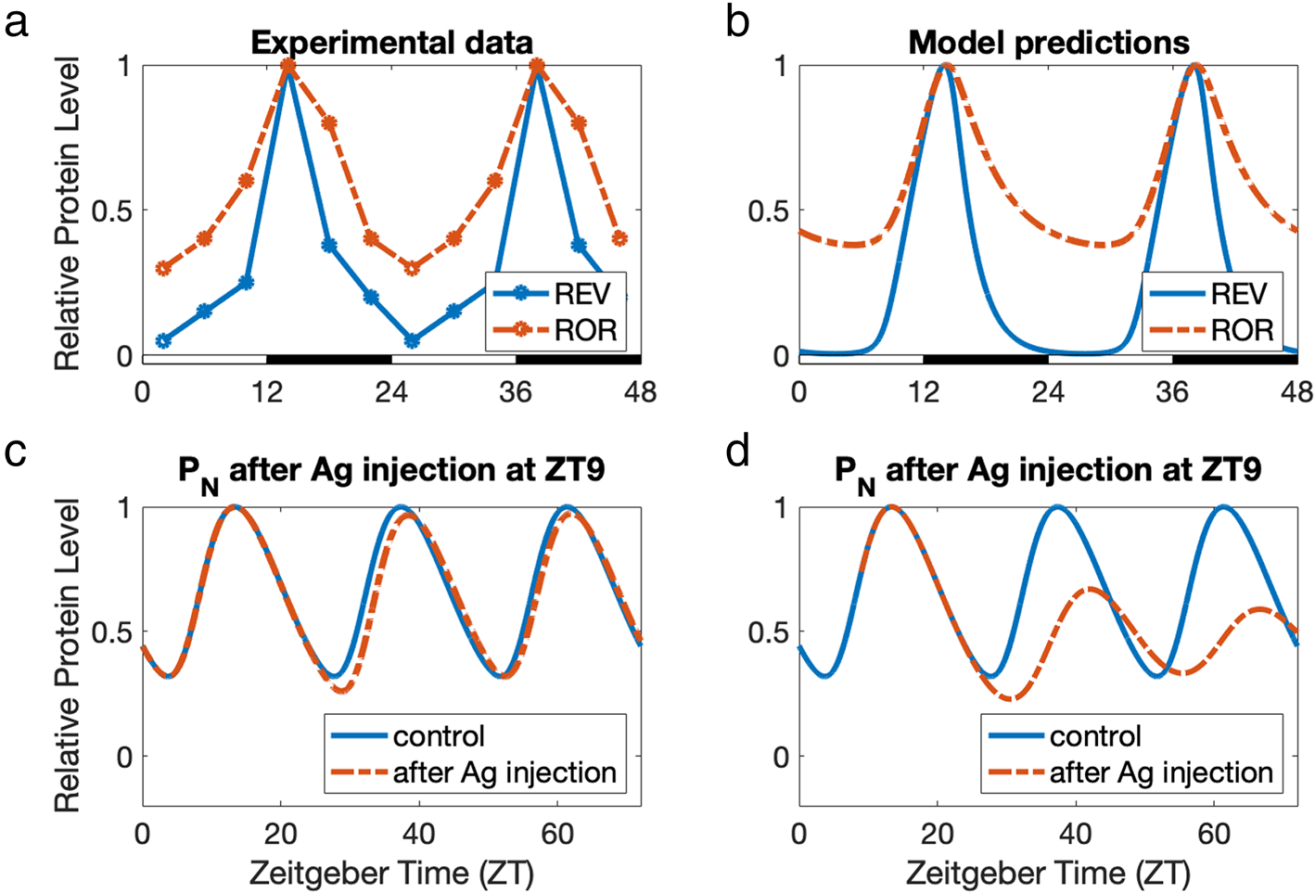
Panels **a** and **b**: REV-ERB and ROR expression levels. **a** Data from Ikeda et al. [12] of REV-ERB and ROR expression levels relative to their peak values in the mouse ventral striatum. Both REV-ERB and ROR peak at around 14 hours Zeitgeber time (ZT14). For REV-ERB, *RMSE*=0.1260. For ROR, *RMSE*=0.1206. **b** Model predictions of *REV* and *ROR* relative to their peak values. We use the same parameter values (Table 2) and set the phase of the model to pertain to the mouse ventral striatum [12], since circadian protein peak times vary across different locations in the body. As in Fig. 4a, *REV* and *ROR* peak at ZT14, with *ROR* dropping to around 40% of its peak value, and *REV* declining to almost zero. *REV* and *ROR* compete to bind to the ROR-response elements (ROREs) in Bmal1, Th, and DRD3 gene promoters. The inhibition by *REV* is thought to be stronger than the activation by *ROR* [12, 44], and in our model, the competition between *REV* and *ROR* creates a net effect of inhibition near the peaks at ZT14 and a net effect of activation away from the peaks. Panels **c** and **d**: Phase-shifting impact of a REV-ERB agonist. The blue curves show the variation of *P*_*N*_ relative to its peak value in the normal model. The dashed orange curves show levels of *P*_*N*_ in the model after injecting *Ag*. **c** Mouse experiments by Solt et al. [11] demonstrate the potential for a REV-ERB agonist that bolsters the effects of inhibition by REV-ERB to aid in the treatments of metabolic and sleep disorders. At a molecular level, injection of a REV-ERB agonist alters the phase of core clock genes. Consistent with Figure 2e of [11], our simulation of a REV-ERB agonist (*Ag*) injection at ZT0 shifts the clock’s phase 1–3 hours to the right for at least 48 hours, even though the agonist has left the system within 24 hours. While Solt et al. [11] measured core clock gene expression levels for 48 hours after injection of a REV-ERB agonist, we ran our model for 120 hours and observed that the clock returns to its light-entrained phase within 2–6 days depending on the amount of agonist. **d** In Figure 2e of [11], only the effects of an injection at ZT0 are considered, as opposed to other injection times. A REV-ERB agonist injection at ZT9 results in a larger initial phase shift in our model, with disrupted circadian rhythms in the long run. This suggests that for certain individuals, circadian rhythms may be sensitive to injection time

**Fig. 5:**
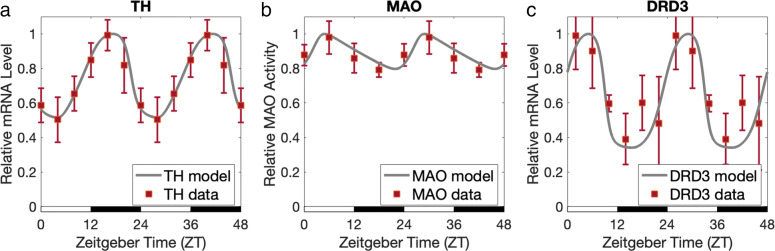
Tyrosine hydroxylase (TH), monoamine oxidase (MAO), and dopamine receptor D3 (DRD3) expression levels. Our model predicts circadian rhythms of TH, MAO, and DRD3 as a result of modulation by clock components REV-ERB (*REV*), ROR (*ROR*), and BMAL1-CLOCK (*BC*) (see Fig. 1). Our model predictions for the expressions levels of TH, MAO, and DRD3 are plotted with gray curves, and the experimental data from [12, 13, 15] are regraphed with red squares. The error bars represent standard deviation. **a** TH is the rate-limiting enzyme in dopamine (DA) synthesis. Consistent with experimental data from [13], TH levels peak at night and drop to around 0.5 of the peak value during the day. *RMSE*=0.05.**b** MAO is essential for the degradation of DA. Consistent with experimental data from [15], MAO levels are nearly antiphasic to TH, and drop to around 0.79 of the peak value. Additionally, the roles of TH and MAO in the DA system, along with their patterns of expression, are consistent with the fact that mice are nocturnal and therefore need more DA at night. It has been confirmed experimentally by Castañeda et al. [16] that DA levels in the rat brain peak during night. *RMSE*=0.04.**c** The dopamine receptor D3 (DRD3) is a DA receptor subtype thought to play an important role in cognition [32]. We model circadian rhythms of DRD3 as a result of repression by REV-ERB and activation by ROR in the mouse ventral striatum, and compare with experimental data from [12]. *RMSE*=0.12

**Fig. 6:**
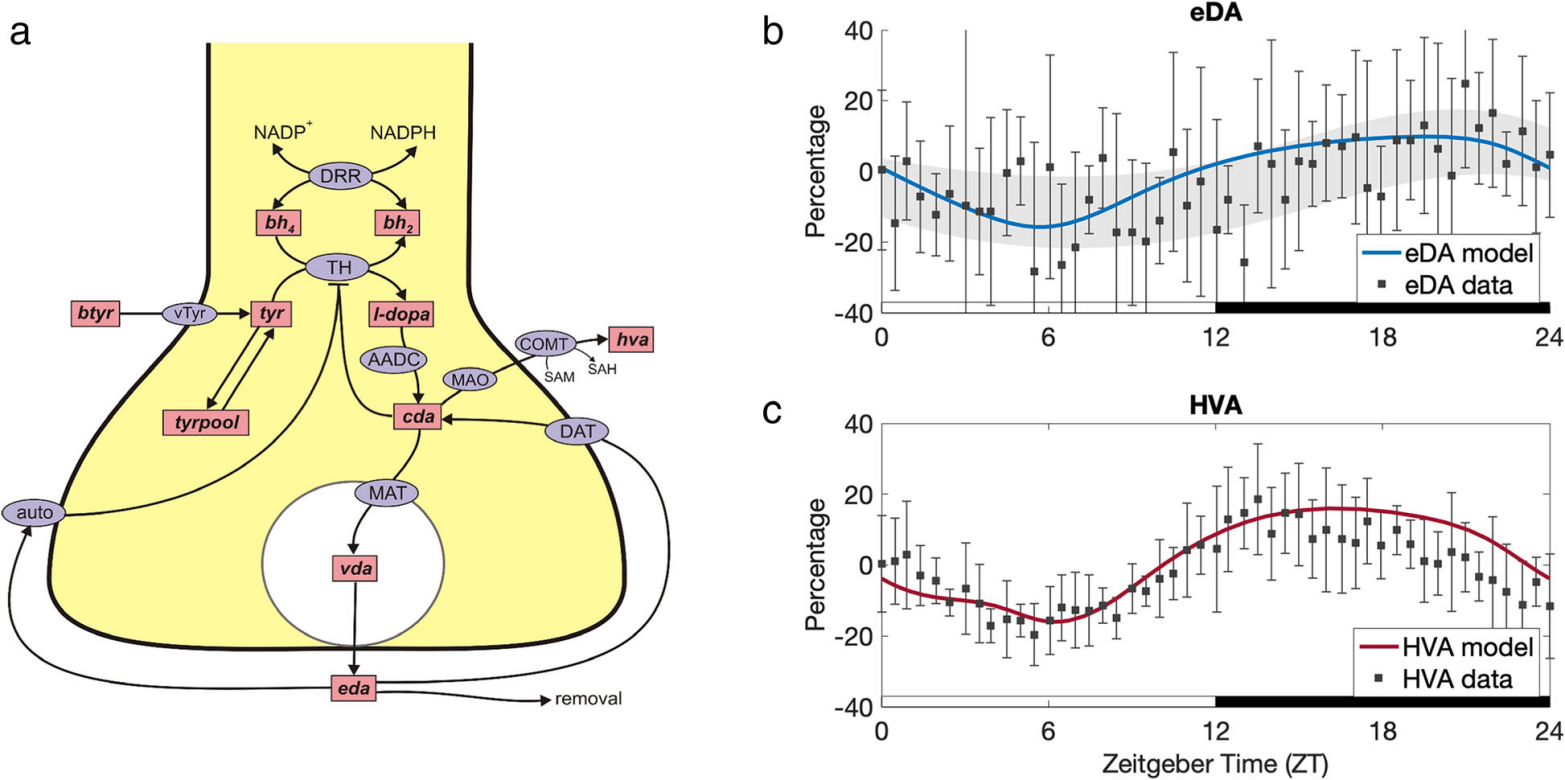
Circadian rhythms of extracellular dopamine (eDA) and homovanillic acid (HVA). The mathematical model in this paper was used to compute the (scaled) expression levels of TH, ETH(t), and MAO, EMAO(t), as functions of time over a 24 hour period, 12 hours light and 12 hours dark. Then, the mathematical model of Best et al. [29] (Panel **a**) was used to compute predicted concentrations of eDA and HVA over the 24 hour period. **a** The DA Model of synthesis, release, reuptake, and control by autoreceptors. Rectangular boxes indicate substrates and blue ellipses contain the acronyms of enzymes or transporters. Full details of the mathematical model are in [29]. Abbreviations: btyr, blood tyrosine; bh2, dihydrobiopterin; bh4 tetrahydrobiopterin; tyr tyrosine; l-dopa, 3,4-dihyroxyphenylalanine; cda, cytosolic dopamine; vda, vesicular dopamine; eda, extracellular dopamine; hva, homovanillic acid; trypool, the tyrosine pool; vTyr, neutral amino acid transporter; DRR, dihydrobiopterin reductase; TH, tyrosine hydroxylase; AADC, aromatic amino acid decarboxylase; MAT, vesicular monoamine transporter; DAT, dopamine transporter; auto, D2 dopamine auto receptors; MAO monoamine oxidase; COMT, catecholamine O-methyl transferase. **b** Predicted time course of eDA concentration compared to data. The model prediction of eDA diurnal variation relative to its average concentration (blue curve) corresponds well to the measurements of Castañeda et al. [16] using microdialysis in the rat striatum. Experimental data from [16] of eDA concentration relative to its average value is plotted with black squares. The error bars represent standard deviation. The gray shaded region outlines the trend of the experimental data; see “Validating the model” for details. Consistent with the data, the model curve of eDA concentration is low during light and high during dark. *RMSE*=10.05*%*.**c** Predicted time course of HVA concentration compared to data. The model prediction of HVA concentration (red curve) also follows the diurnal pattern observed in experimental data from [16], plotted with black squares. The error bars represent standard deviation. The model curve is almost entirely within one standard deviation of each data point. HVA concentration drops to about 16 percent below its average value during light, and about 16 percent above during dark. *RMSE*=6.38*%*. The results shown in Panels **b** and **c** give strong confirmation that the REV and ROR mechanisms reviewed in [20] and investigated in [12, 13, 15] are sufficient to explain the effects of the circadian clock on the dopaminergic system

For us, validating the model means comparing its predictions to a large set of different experimental results from different laboratories on different parts of this complex system. In Figs. 2 and 3 we present model experiments that show that the model results reproduce key features of the circadian clock by giving references to the literature. In Figs. 4, 5, and 6, we compare our model predictions directly to experimental data on the secondary feedback loop, the downstream influences, and the dopaminergic variables. The experimental data in Figs. 4 and 5 were used in our choices of parameters but the data in Fig. 6 was not, so Fig. 6 is a test of the model.

In “Results,” Figs. 3, 4, and 5 show our model predictions with the y-axis as relative protein or mRNA level. This is the expression level of the corresponding protein or gene relative to its peak expression level, throughout the day. We display our results in this way because the experimentalists display their data in this way. Figures 6b and 6c display our model predictions with the y-axis as concentration relative to the average value, as this is how the experimental data is presented. In all cases, we show the standard deviation of each experimental data point. Figure 6b is a special case because the data is so variable that it is hard to see the trend visually. So, we plot a gray shaded region computed as follows. At each of 5 equally spaced experimental data points, we computed the average value across the previous 3 data points and following 3 data points, as well as their average standard error of the mean. We then used the MATLAB spline function to create an average standard error region across a 24 hour period. In addition, for each of the comparisons with data, we compute the root mean square error (RMSE) as follows:

31 $$ RMSE = \sqrt{\frac{1}{n}\sum_{i=1}^{n} (x_{i} -y_{i})^{2}}  $$

where *n* is the number of data points, the {*y*_*i*_} are the experimental data points, and the {*x*_*i*_} are the corresponding model points.

## Results

### The core circadian clock

We refer to the variables *BC*, *P*_*i*_ where *i* is the number of phosphorylations, *C*, *PC*, *PC*_*N*_,*P*_*N*_,*C*_*N*_, as **the core circadian clock**; see Fig. 1. As explained in the “Methods,” our core oscillator is an extension of the core oscillator model developed by Kim and Forger [26, 27]. Our oscillator additionally incorporates the binding of PER and CRY in the cytosol to enable nuclear entry of CRY, and the dissociation of PER-CRY in the nucleus to enable strong repression of BMAL1-CLOCK-mediated transcription by CRY alone [17, 18]; see “Methods.”

In the mouse core oscillator in the superchiasmatic nucleus (SCN), the binding of the proteins BMAL1 and CLOCK in the nucleus (BMAL1-CLOCK) promotes the transcription of Per and Cry genes during light. The PER and CRY proteins are transported to the cytosol, where PER is phosphorylated and then binds to CRY, and the PER-CRY complex is transported back into the nucleus. The PER-CRY complex in the nucleus binds to BMAL1-CLOCK at the beginning of dark when nuclear PER-CRY peaks, to repress BMAL1-CLOCK through protein sequestration [37, 38]. The PER-CRY complex also dissociates in the nucleus and CRY inhibits BMAL1-CLOCK-mediated transcription strongly [17, 18]. The result of this feedback inhibition loop can be seen in Fig. 2.

In our model, cytosolic PER (*P*_1_) and cytosolic CRY (*C*) peak shortly after BMAL1-CLOCK (*BC*) (Panels A, B, and D). The delays between cytosolic PER and nuclear PER (*P*_*N*_), and between cytosolic CRY and nuclear CRY (*C*_*N*_), are around 9 hours (Panels A and B). Nuclear PER and CRY reach their peak values at the beginning of dark, around 12 hours Zeitgeber time (ZT12)^1^, consistent with data described in [38]. Nuclear PER and nuclear CRY bind and form nuclear PER-CRY (*PC*_*N*_), which binds to and represses BMAL1-CLOCK in our model. Thus, when nuclear PER-CRY peaks at ZT12, the expression levels of cytosolic PER and CRY are significantly reduced. Additionally, nuclear CRY strongly inhibits the production of cytosolic PER and CRY during night, adding another mode of repression in the circadian feedback loop. Ye et al. [17] propose this model of the core oscillator, and while the exact function of these dual modes of repression are yet unknown, they could be investigated using our model in future work.

Our core oscillator responds correctly to light-dark. As described in “Methods,” we model the production of PER to be dependent on light, as it is known that Per gene expression is light-induced [34–36]. Solt et al. [11] measured the expression levels of core clock genes in the hypothalamus under 12hr light 12hr dark conditions, as well as in constant darkness for mice previously entrained to a 12hr light 12hr dark cycle. In constant darkness, the expression of Per2, a polymorphism of the Per gene, was still rhythmic, but greatly reduced in comparison to light-dark data [11, Figure 2c]. Our model (see Fig. 3) agrees with this dampened expression, and displays a slightly shorter circadian period length in constant darkness. The period length of mouse circadian rhythms without light input is known to be shorter than 24 h (23.5 h) [39].

Our model also confirms disruption in circadian rhythmicity as a result of CRY deficiency. Although our focus is on downstream dopaminergic variables, we chose to include the effects of CRY deficiency because our model introduces a new repression term dependent on CRY; see “The core circadian clock” section. Studies by van der Horst et al. [40] and Vitaterna et al. [41] in the mouse SCN have shown that CRY null mutant mice display nearly diurnal oscillations and elevated PER expression levels under 12hr light 12hr dark conditions. In CRY null mutant mice under constant darkness, circadian rhythmicity is immediately disrupted, with approximately constant PER expression levels [41]. While the experiments in [40] and [41] study the separate effects of mutations in different polymorphisms of CRY, we confirm that our model predicts the general qualitative behavior of CRY deficiency on circadian rhythms under 12hr light 12hr dark versus constant darkness. We model CRY deficiency by reducing the production rate of CRY by 95%. This results in nearly diurnal oscillations and elevation in the expression of cytosolic PER (*P*_1_) under 12hr light 12hr dark conditions, consistent with the experimental observations in [40, 41] described above; see Fig. 3a. With CRY deficiency under constant darkness, our model predicts a disruption of circadian rhythms and near-constant variation of *P*_1_, also consistent with [40, 41]; see Fig. 3b.

### REV-ERB and ROR

The transcriptions of orphan nuclear receptor REV-ERB (*REV*) and retinoic acid-related orphan nuclear receptor ROR (*ROR*) are activated by BMAL1-CLOCK (*BC*). In turn, they affect the circadian clock because they modulate the transcriptional activity of the Bmal1 gene [10, 42, 43]. *REV* inhibits and *ROR* activates the transcription of Bmal1 and this feedback loop is thought to improve the homeostasis of the clock. The experiments by Ikeda et al. [12] show that *REV* and *ROR* have 24 hour rhythms and both peak at approximately ZT14 in the mouse ventral striatum; see Fig. 4a in which we have regraphed the data from [12].

In our model, the influence of *REV* and *ROR* on *BC* is through an intermediary step, *S*, which can be thought of as Bmal1; see the Methods and Fig. 1. *REV* inhibits *S* and *ROR* activates *S*. We use the same parameter values (Table 2) and set the phase of the model to pertain to the mouse ventral striatum [12], since circadian protein peak times vary across different locations in the body. Figure 4b shows that the model curves for *REV* and *ROR* closely predict the experimental curves in Fig. 4a. Not only do *REV* and *ROR* peak together at ZT14, but the *REV* peak is also narrower than the *ROR* peak, as in the experimental data. Also consistent with [12], the *REV* curve declines almost to zero while the *ROR* curve declines to 40% of its peak value. It is thought that the inhibition by *REV* is stronger than the activation by *ROR* [12, 44], so near the peaks at ZT14 the net effect is inhibition, while away from the peaks the net effect is activation. As we will see below, it is the competition between *REV* and *ROR* that controls the dopaminergic variables.

Solt et al. [11] developed a potent synthetic REV-ERB agonist (SR9011) and conducted experiments in which the agonist was injected into mice at ZT0. The experiments demonstrated potential for the agonist to aid in the treatment of metabolic diseases and sleep disorders. On the molecular level, the REV-ERB agonist injected at ZT0 delayed the phase of core clock gene expression levels in the hypothalamus by 1–3 hours for 48 hours, graphed in Figure 2e of [11].

In our model, the variable *Ag* represents a REV-ERB agonist that increases *REV*-dependent repressor activity and decays within 24 hours, as in [11]. Consistent with experimental results, an injection of *Ag* in the model at ZT0 shifts the clock’s phase 1–3 hours to the right for at least 48 hours though the agonist leaves the body within 24 hours of injection. Though all clock components shifted by the same amount, we graph just *P*_*N*_ in Fig. 4c-d for clear visualization. In [11], clock gene expression levels were measured for 48 hours after injection, and the agonist was administered only at ZT0 (Figure 2e in [11]).

Our model predicts that the phase shifting effect of a REV-ERB agonist at ZT0 is temporary, and that injection time is important. When we run our simulations beyond 48 hours after injection, our clock reverts back to its original phase in 2–6 days; see Fig. 4c. This makes sense because the circadian phase is entrained to light. For someone with an early circadian phase, maybe due to jet lag, a REV-ERB agonist-induced phase shift may aid with quicker entrainment to the light-dark cycle.

Additionally, we experimented with different injection times. Figure 4d depicts *P*_*N*_ after an injection at ZT9. While the initial shift in clock components for a REV-ERB agonist injection at ZT0 is about 1–3 hours with a return to its original light-entrained phase after 3 days, a REV-ERB agonist injection at ZT9 results in the complete disruption of circadian rhythmicity. This suggests that for certain individuals, circadian rhythms may be sensitive to injection time. We note that in our model, there exist other sets of parameter values that result in reduced sensitivity to the agonist. Additionally, the phase-shifting impact of a REV-ERB agonist is dose-dependent, being more prominent at higher doses of *Ag*. Supported by our model, we propose that strategic timing and dosage could impact the physiological effects of a REV-ERB agonist, including treatment of metabolic diseases and sleep disorders as discussed in [11].

### DA system

The dopamine (DA) system is known to play an important role in movement, reward, and memory, among many other physiological functions [1, 2, 5], and DA system dysfunctions have been linked to Parkinson’s, mood disorders, and schizophrenia [4, 6]. Several studies have measured the circadian rhythms of tyrosine hydroxylase (TH) and monoamine oxidase (MAO), key components of the DA system [13–15, 20].

TH is the rate-limiting enzyme in DA synthesis. Chung et al. [13] measured TH mRNA levels in the mouse midbrain, as well as TH protein expression levels in the ventral tegmental area (VTA) and substantia nigra (SN) in the midbrain, and showed that TH mRNA and protein levels in mice previously entrained to a 12hr light 12hr dark schedule^2^ peak at night and dip during the day, similarly to Bmal1. Consistent with the data, *TH* in our model peaks at night and drops to around 0.5 of its peak value; see Fig. 5a. Rhythms of core clock genes such as Bmal1 and Per2 in mouse midbrain data from Figure 2a of [13] display similar diurnal variations to mouse SCN data, which we’ve previously used to check our model (Fig. 2). As a result, we used the same parameter values as in “The core circadian clock.”

MAO activity, which is involved in the degradation of DA, was measured in the VTA and ventral striatum (NAc) by Hampp et al. [15]. MAO-a (one of two isozymes of MAO) mRNA followed circadian rhythms, reaching its peak at around ZT6 and dropping to around 0.79 of its peak value in the VTA and ventral striatum, which is what our model predicts (Fig. 5b).

The dopamine receptor D3 (DRD3) is a DA receptor subtype thought to play an important role in cognition in both healthy individuals and those with neuropsychiatric disorders, such as Parkinson’s disease and Alzheimer’s disease [32]. In “REV-ERB and ROR,” we looked at REV-ERB and ROR data from Ikeda et al. [12]. The experiments highlighted measurements of DRD3 mRNA levels in the mouse ventral striatum. Since circadian rhythms vary across different locations, we used our phase adjustment from “REV-ERB and ROR” to compare our DRD3 predictions with [12]; see Fig. 5c.

Our model gives the correct qualitative behavior of downstream DA elements TH, MAO, and DRD3. The fact that MAO in the mouse brain peaks during light and drops during dark, being out-of-phase with TH, is expected. Data by Castañeda et al. [16] of DA in the striatum and nuclues accumbens demonstrate DA diurnal variation in rats, with lower levels during the day and higher levels at night. This makes sense for nocturnal animals that would require DA for motor and physiological functions during night when they are active.

### Effect on extracellular dopamine and homovanillic acid

As we discussed above, it makes physiological sense that the expression level of TH is low and the expression level of MAO is high during light when mice sleep and the converse during dark when mice are active. But how does the diurnal variation in TH and MAO affect extracellular dopamine (eDA) and the breakdown product of DA, homovanillic acid (HVA)? We use the extant mathematical model of DA synthesis, release, reuptake and control by autoreceptors created by Best et al. [29] to predict the effects of the circadian clock on the concentrations of eDA and HVA and compare our predictions to rat striatum data of Castañeda et al. [16].

The model in this paper has as output the expression levels of TH and MAO as functions of time over a 24 h period. As described in “Methods,” we used these functions to compute the time courses of all the variables in the model by Best et al. [29]. Before computing these functions, we adjusted four parameters: *α*_*th*_=3.7,*b*_*th*_=0,*ε*_*th*_=0.3, and *d*_*m*_=0.02. These parameter adjustments are completely appropriate, since variation across different animals and regions of the brain is expected. The predicted concentrations of eDA and HVA are shown by the curves in Fig. 6b-c. The data points are regraphed from the experiments by Castañeda et al. [16] who studied the concentrations of eDA and HVA in the striatum and nucleus accumbens in awake rats using microdialysis. DA in the nucleus accumbens is known to be released from the terminals of neurons in the midbrain [16].

For eDA, there is a lot of noise in the data, but our predicted curve follows the pattern seen in the experiments, with eDA expression levels dropping to 10–15% below the average value during light, and increasing to 10–15% above the average value during dark. The gray shaded region in Fig. 6b outlines the diurnal variation of the experimental data. The predicted time course of eDA from our model lies inside this region, which was computed using average value and standard error at ZT0, ZT6, ZT12, ZT18, and ZT24; see “Validating the model” for details. For HVA, the data is quite regular, and our predicted curve follows the data closely; see Fig. 6c. Consistent with rat striatum data [16], HVA drops to about 16% below its average value during light and about 16% above its average value during dark. These results give strong confirmation that the REV-ERB and ROR mechanisms reviewed in [20] and investigated in [12, 13, 15] are sufficient to explain the effects of the circadian clock on the dopaminergic system.

## Discussion

We have created a mathematical model of mammalian circadian rhythms and their influences on the dopaminergic system. Our model comprises three interlocking systems: the core circadian clock, the secondary feedback loop, and the downstream influences on the dopaminergic system. In “The core circadian clock,” we showed that our model is consistent with major known properties of core circadian genes and proteins. In particular, our core circadian clock model predicts diurnal patterns of expression, and correct responses to light-dark and CRY deficiency in the superchiasmatic nucleus (SCN). In “REV-ERB and ROR,” we showed that our model of the secondary feedback loop containing REV-ERB and ROR is consistent with experimental data on REV-ERB and ROR expression levels in the mouse ventral striatum. We also showed that our model correctly predicts the phase shifting effect of a REV-ERB agonist on the circadian clock. In “DA system,” we showed that our model is consistent with experimental data on the expression levels of dopaminergic variables: tyrosine hydroxylase (TH) and monoamine oxidase (MAO) in the mouse midbrain, and dopamine receptor D3 (DRD3) in the mouse ventral striatum. Finally, in “Effect on extracellular dopamine and homovanillic acid,” we connected our model predictions of TH and MAO circadian variation to the model of DA synthesis, release, and reuptake by Best et al. [29], and showed that the predicted concentration levels of extracellular dopamine (eDA) and homovanillic acid (HVA) agree reasonably well with the experimental data on eDA and HVA in the experiments of Castañeda et al. [16]. Our calculations show that the mechanisms proposed by experimentalists by which REV-ERB, ROR, and BMAL1-CLOCK influence the DA system are sufficient to explain the circadian oscillations observed in dopaminergic variables.

We chose to model the influence of the circadian clock on the dopamine system by using a system of differential equations, because we and the experimental community are interested in the dynamic behavior of the circadian and DA systems and how their dynamics is affected by gene polymorphisms, changes in light input, and biochemical interventions. Such dynamic behavior is much harder to model by using discrete models such as Boolean models. The first step in creating the model was to create a schematic diagram, based on the findings and speculations of experimentalists about what variables are important and how they influence each other. So, the schematic diagram (Fig. 1) is based on the underlying biology. However, the schematic diagram is not itself a mathematical model. To make the mathematical model, one must choose kinetics for each of the “influences,” and in most cases there is little experimental evidence on the kinetics. So, we chose relatively standard forms for the kinetics (mass-action, Michaelis-Menten) that have been used in other biochemical modeling where kinetics is known. Of course, the kinetic formulas have parameters and we chose them by trial and error so that the model predictions qualitatively predict behaviors seen in a large number of different experiments by different laboratories on different aspects of this large and complicated system.

Often the experimental data is sparse and variable, consisting of five or six data points over 24 hours, each calculated as the average over a small number of animals. One can see the standard deviations in Figs. 4, 5, and 6. So, it doesn’t make sense to go to great lengths to choose parameters so that the model “fits” any particular data set. In fact, the situation is even more difficult. We made our model to be a platform for investigating the mechanisms by which the mammalian circadian clock affects the DA system. Certainly, the parameters of the model will vary depending on the species and on the brain region being examined. Furthermore, it is known that enzyme expression levels in the same locations vary by about 25% between individuals of the same species [45–47]. What this means is that there is no “correct” set of parameters. In this situation, validation of the model means that its predictions correspond qualitatively with a large amount of data on different aspects of the system collected by many different labs. In Fig. 2 and 3 we show only model predictions but give references so the reader can compare to data in the literature. In Figs. 4, 5, and 6, the main goal of the paper, we present different data sets and calculate how close our predictions are to the data. No model can represent the full complexity of the biological situation, but we believe that our model represents reasonably well the underlying biology and can be used for investigating biological hypotheses about the mechanisms by which circadian rhythms affect the dopaminergic system.

We have focused on the mechanisms by which circadian rhythms affect the dopaminergic system. We have used the model to explore some effects of light-dark and a CRY gene mutation, but much more experimental information is available on light-dark, gene mutations, and gene polymorphisms that could be explored and interpreted in future work. There is also a large literature on how variations in the dopaminergic system affect behavioral variables and neuropsychiatric diseases [1–6]. To bridge the gap between our mechanistic model and behavioral variables, one needs to use model reduction techniques such as those in [48].

Several experimental studies have shown that disruptions to the core circadian clock alter the dopaminergic system. Mice with a knock-out mutation for Per1, a polymorphism of the Per gene, displayed ADHD-like behavior and reduced levels of DA [49]. Mice with a mutation in the Clock gene demonstrated an increase in dopamine cell firing in the ventral tegmental area (VTA) [50]. Our mathematical model can be used to study the effects of circadian disruptions on the dopaminergic system, and potentially be used to find drug targets.

There is a large amount of experimental evidence that the dopaminergic system itself affects the circadian clock. DA depletion in non-human primates disrupts locomotor rhythms in different light-dark conditions [51]. In the rat forebrain, DA depletion has been shown to reduce PER2 expression levels [52, 53]. The studies in [53] also suggest that the activation of particular DA receptors helps to restore PER2 rhythms in the DA-depleted rat striatum. Augmenting the model presented in this paper to include the influence of dopaminergic variables on the circadian clock will be the subject of future work.

## Conclusions

We have created a mathematical model for the core circadian clock, the secondary feedback loop, and the downstream influences on the dopaminergic system. Our calculations give strong confirmation that the REV-ERB, ROR, and BMAL1-CLOCK mechanisms reviewed in [20] and investigated in [12, 13, 15] are sufficient to explain the effects of the circadian clock on the dopaminergic system. Our mathematical model can be used for further investigations of the effect of the mammalian circadian clock on the dopaminergic system. The model can be used to predict how perturbations in the circadian clock disrupt the dopamine system and could potentially be used to find drug targets that ameliorate these disruptions.

## Data Availability

The complete mathematical model is described in detail in the “Methods” section. The code for the mathematical model is available from the authors.

## Footnotes

1 Zeitgeber time (ZT) is a unit of time corresponding to the 24-hour light-dark cycle, with ZT0 indicating the beginning of light and ZT12 indicating the beginning of dark.

2 In [13], lights on was at 8 h Circadian Time (CT8), or ZT0 in our model.
